# AAPM DWWSS report 449: Evaluation of imaging physics workforce, workload, and future needs in the United States

**DOI:** 10.1002/acm2.70843

**Published:** 2026-10-06

**Authors:** Joseph R. Steiner, Shengwen Deng, Ching‐Mei Feng, Joshua M. Wilson, Mary Ellen Jafari, Megan E. Lipford, Lei Qin, Dustin Gress, David Jordan

**Affiliations:** ^1^ Department of Radiology University of Chicago Chicago Illinois USA; ^2^ Department of Radiology University Hospitals Cleveland Medical Center, Case Western Reserve University School of Medicine Cleveland Ohio USA; ^3^ Department of Diagnostic and Interventional Imaging UTHealth McGovern Medical School Houston Texas USA; ^4^ Clinical Imaging Physics Group Duke University Health System Durham North Carolina USA; ^5^ Department of Radiology Duke University Durham North Carolina USA; ^6^ Southern California Permanente Medical Group Pasadena California USA; ^7^ Wake Forest University School of Medicine Winston Salem North Carolina USA; ^8^ Dana‐Farber Cancer Institute Boston Massachusetts USA; ^9^ American College of Radiology Reston Virginia USA

**Keywords:** diagnostic medical physics, nuclear medical physics, workforce

## Abstract

**Introduction:**

In 2020, the American Association of Physicists in Medicine (AAPM) conducted a comprehensive imaging physics workforce survey (2020 Survey) to evaluate staffing needs, validate prior time and effort estimates, and explore workforce satisfaction. This report summarizes key findings, highlighting gaps between training and professional demands, challenges in recruitment, and shifts in workforce demographics and employment patterns.

**Methods:**

The 2020 Survey, administered by the American Institute of Physics (AIP) Statistical Research Center (SRC) to AAPM and Health Physics Society members, had 953 responses from participants who were primarily diagnostic and nuclear medical physicists. The data were analyzed across four key areas: professional task workloads, workforce demographics and retirement outlook, lifestyle factors, and skill gaps.

**Results and Discussion:**

The AIP SRC analyzed the results of the 2020 Survey (results archived on the AAPM members‐only website). The 2020 Survey validated the relative time required for equipment performance evaluations and the wide range of Level 2 services performed by the imaging physics workforce relative to AAPM Report 301. The 2020 Survey showed that the imaging physics workforce is aging (30% of respondents providing their age stated they were 66 years or older) and that many of these individuals could retire within 5 years of completing the survey. Finally, the 2020 Survey demonstrated that there were gaps between the skills and knowledge employers look for when hiring compared to the same self‐identified qualities of the imaging physics workforce.

**Conclusions:**

The 2020 Survey provided insights into the imaging physics workforce that validated prior publications estimating imaging physics workforce activities. The 2020 Survey indicates the imaging physics workforce is aging, with a wave of retirements on the horizon, and that individuals entering the workforce may not possess the skills and knowledge desired by employers. The data from this survey can be used to guide analyses and address future trends of the imaging physics field and workforce.

## 1 INTRODUCTION

The continued stability and viability of the medical physics profession depends on a reliable, accurate understanding of the current state of the workforce as well as the ability to anticipate and influence projected trends. Historically, efforts to study the imaging physics workforce and needs have been divided into two distinct categories: 1) surveys of the existing imaging physicist workforce that characterize their demographics and work^1^; and 2) expert analysis (imaging physicists working as committees) and surveys of imaging physicists to estimate the amount of time needed to perform tasks, which can then be used to estimate FTE load.^2^, ^3^, ^4^

The imaging physics workforce, including the subspecialties of diagnostic medical physics and nuclear medical physics, has been studied twice in efforts led by the American College of Radiology (ACR)^1^ and American Association of Physicists in Medicine (AAPM).^2^ Due to the rapid change in technology, training requirements, and how healthcare is practiced, previous comprehensive publications on the imaging physics workforce may have limited relevance to the current workforce, which highlights the need for an updated assessment.

In 2017, the AAPM Diagnostic Workforce Subcommittee (DWWSS) published AAPM Report 301^3^ with the goal of framing questions and issues needed to understand imaging physics workforce and staffing questions in the modern healthcare setting. That report set forth definitions and terms for typical professional duties in imaging physics, such as annual Equipment Performance Evaluations (EPEs). The report included detailed estimates of time and effort requirements for annual EPEs that were determined by expert consensus of experienced imaging physicist members of DWS and benchmarked against previously published data.^1^

AAPM Report 301 also introduced the levels of service (LoS) model as a practice‐model‐independent approach to determining staffing needs based on the requested medical physics services and the corresponding time and effort required. The LoS model marked a departure from prior assessments that had arrived at different time and effort findings for the varied imaging physics practice‐models such as in‐house employed physicists and consulting or contract physics service providers. As a result, the LoS model ascribes the differences commonly observed between practice environments as functions of the amount and type of services a given facility needs and wants, rather than as inherent characteristics of how the imaging physicists providing those services received their wages (e.g., in‐house or consultancy/contract). Defining the EPE and LoS paradigms was done in anticipation of future surveys to collect and analyze effort data without partitioning the workforce by employment setting.

In 2018, DWWSS partnered with the American Institute of Physics (AIP) Statistical Research Center (SRC) to develop and administer to the AAPM membership a preliminary survey entitled “Trends in Diagnostic and Nuclear Medicine Physics” (2018 Survey). This survey was designed to provide data to answer questions raised by anecdotal observations, including a perceived cresting wave of retirements and difficulties in recruiting qualified candidates for job vacancies in imaging physics. Over 1700 AAPM members responded to the survey. The demographics of respondents (i.e., highest degree, completion of accredited programs, certification, and gender identity) were consistent with the typical findings of AAPM's annual professional survey. Respondents involved in hiring reported their primary reasons for hiring difficulty. These findings were used to guide the development of the present workforce survey.

DWWSS used the results of these predicate efforts to develop the “AAPM Diagnostic Workforce Survey” (2020 Survey), which was administered in late 2020 to members of the AAPM and Health Physics Society (HPS). The primary goal of the 2020 Survey was to improve understanding of the current workforce; this ranged from workforce demographics to time required to perform work‐related tasks to a self‐assessment of personal skills and lifestyle factors. The 2020 Survey was a comprehensive survey of the imaging physics workforce with four aims:

1. Validate EPE workloads of imaging physicists, primarily time spent performing these tasks (specifically, to validate Level 1 services^3^).
2. Measure the time and effort dedicated to common professional responsibilities of imaging physicists beyond performing EPEs (specifically, Level 2 services^3^).
3. Assess the prevalence of workforce age and lifestyle factors that could adversely affect job satisfaction, recruitment, retention, attrition and retirement for imaging physicists in the United States.
4. Evaluate gaps and disconnects in between the imaging physics workforce educational and training background and the skills and knowledge required or desired for the professional roles of its members, as perceived by both imaging physicists and their employers.

This report summarizes the key findings of the 2020 Survey and discusses their interpretation and potential impacts.

## 2 METHODS

The 2020 Survey was developed by DWS and the AIP SRC. The survey was voluntary, was designed to be completed in approximately 30 min, and was administered on behalf of the AAPM by the AIP SRC to members of the AAPM and of the Medical Health Physics Section (MHPS) of the HPS. 50% (3230 members) of the AAPM full membership list was randomly selected regardless of indicated primary specialty (or none listed) to receive the 2020 survey. 100% (490 members) of the HPS MHPS full member list were selected to receive the 2020 survey. The recipient pool was not pre‐selected in any way, although based on the survey questions and self‐selection it was expected there would be a high proportion of imaging physicists in the responses. 953 responses were collected, providing a response rate of 26%. Of these responses, 795 were AAPM members and 158 were HPS members. Of the 795 AAPM member responses, 98 respondents indicated in response to a screening question that they were not diagnostic physicists, did not have time to complete the survey, or were retired. These 98 respondents did not see the remaining survey questions, and thus 697 AAPM members responded to the questionnaire. The AIP summary of the 2020 Survey results and the survey questionnaire are archived on the AAPM members‐only website (https://www.aapm.org/pubs/surveys.asp).

The DWS reviewed and evaluated the results of the 2018 and 2020 Surveys, especially the analyses provided in the AIP's summary, to gain specific insights about the diagnostic and nuclear medical physics workforce. This report serves to highlight results the DWS found noteworthy, specifically related to the four aims identified in the introduction.

## 3 RESULTS AND DISCUSSION

### 3.1 EPE time and effort estimates

EPEs are Level 1 service surveys or testing of imaging equipment performed by medical physicists or other qualified individuals.^3^ Previous reports from 2004^1^ and 2017^3^ provided estimates of time required to perform annual EPEs for various imaging equipment. The 2020 Survey provides updated data to validate the time estimates published in these two prior reports (Table 1).

**TABLE 1:** Time to perform annual imaging EPEs. Data from Table 11 of the 2020 survey, Table 1 of AAPM Report 301,^3^ and Tables 3 and 4 of the Cypel and Sunshine report.^1^

	Average number of hours per		
Modality	2020 survey^*^	AAPM Report 301^**^	ACR report^***^
MQSA‐compliant FFDM with DBT	4.9	8	8 (mammography tubes)
CT	3.9	4	6
Radiography	1.7	2	3 (excluding portables)
Conventional fluoroscopy or mobile C‐arm	1.6	3	3
Interventional fluoroscopy, angiography or Cath lab	2.6	6	Not provided
CT subsystem of PET/CT	3	Not provided	Not provided
CT subsystem of SPECT/CT	3	Not provided	Not provided
SPECT or dual‐head gamma camera	4.8	8	8
PET including PET subsystem of PET/CT or PET/MRI	3.8	6	37
Ultrasound	1.5	2	3
MRI including MRI subsystem of PET/MRI	5.3	8 (for standalone MRI only)	9 (for standalone MRI only)

Abbreviations: CT, computed tomography; DBT, digital breast tomosynthesis; FFDM, full field digital mammography; MQSA, mammography quality and standards act; MRI, magnetic resonance imaging; PET, positron emission tomography; SPECT, single photon emission computed tomography.

* from 2020 Survey, values are for hands‐on equipment testing only, only for full‐time respondents performing these surveys and with outliers removed. All data (including data without removing outliers) can be accessed at the AAPM survey webpage https://www.aapm.org/pubs/surveys.asp

** from AAPM Report 301,^3^ values include time for required tasks other than hands‐on equipment testing (e.g., report generation, QC program review, protocol review) without modality‐specific modifiers that capture follow‐up testing events.

*** from Cypel and Sunshine,^1^ values reported are mean values for workers in diagnostic medical physics only.

The 2020 Survey results for hands‐on time to perform EPEs are similar to or less than the previously published values across all modalities. This was an expected result as the EPE time requirements in AAPM Report 301 included additional tasks (e.g., report preparation, QC program review, protocol review), whereas the 2020 Survey focused narrowly on the time required for the medical physicist to perform the hands‐on testing portion of the EPE. The 2004 ACR survey^1^ did not specify the components of each evaluation or how survey respondents were directed to quantify time. The hands‐on time reported in the 2020 Survey is valuable for informing clinicians on required equipment down time to perform EPEs whereas the data reported in AAPM Report 301 is more useful in identifying the total effort spent on a given EPE by an imaging physicist. Both of these results (2020 Survey and AAPM Report 301) may be used to further verify the current imaging physicist FTE models developed by IAEA^4^ or to develop new imaging physicist FTE models.

The 2020 Survey reflects the same hierarchy of time demands across different diagnostic imaging and nuclear medicine equipment as AAPM report 301^3^. The times reported for magnetic resonance imaging (MRI), computed tomography (CT), positron emission tomography (PET), single photon emission computed tomography (SPECT) and mammography evaluations are higher than other modalities. This is consistent with the authors' experience that EPEs for these modalities are generally more demanding, due to the complexity of the modality itself and/or the comprehensive testing required to meet accreditation and/or regulatory standards. This observation supports commentary in AAPM report 301, which recognizes the additional testing and compliance considerations associated with advanced imaging modalities.

The time requirements from the ACR study^1^ were largely similar to those in AAPM Report 301,^3^ and both are consistent with the findings of the 2020 Survey. Considering the increased complexity of imaging equipment and technology in the intervening years between these studies, such as widespread adoption of digital breast tomosynthesis (DBT), this is an unexpected finding. An increase in the complexity of imaging EPEs (including associated tasks) without an associated increase in time required to perform the EPEs may suggest that the imaging physics workforce is becoming more competent and efficient, potentially due to the maturation of the field, such as 1) improved training models,^5^, ^6^ 2) more stringent requirements relating to gaining eligibility for board certification, 3) evolving imaging modalities (e.g., digital detectors in radiography reduce added time/complexity relative to screen film and phosphor plates), 4) more sophisticated evaluation instruments (e.g., using a solid‐state dosimeter to measure half‐value layer in radiography as opposed to an ion chamber and aluminum sheets), 5) automation (e.g., direct connection of measurement devices to portable computers and increased features in word processing and spreadsheet software), and 6) a possibility that manufacturers may have improved or streamlined dedicated physics testing protocols on their systems. An alternative possibility is that the procedures and time requirements for EPEs were well‐established in imaging physics practice by 2004, anchoring expectations such that imaging physicists adapted to subsequent developments by fitting testing procedures into accepted and allotted time slots. This alternative possibility can be supported by structured activities for minimal level of clinical support detailed in medical physics practice guidelines (Level 1 activities), versus the more extensive Level 2 and 3 testing for optimal equipment performance recommended by task group reports.

The EPE tasks in addition to the hands‐on testing reported in AAPM Report 301 (e.g., report generation, QC management, protocol review), may require a few minutes up to several additional hours for each EPE, depending on modality as based on the collective experience of the DWS. When accounting for this difference, the 2020 Survey results agree with the imaging EPE times reported in AAPM Report 301. There is a strong alignment between the theoretical framework outlined in AAPM Report 301, the real‐world data from the DWS 2020 Survey, and the qualitative insights from the 2004 ACR study. This convergence strengthens the validity and practical applicability of the structured model for medical physicists' services, enabling better resource planning and workload estimation across various diagnostic imaging modalities. The consistent trends and relative time demands across modalities reinforce the practical applicability of the AAPM Report 301 framework, while also highlighting modalities where further updates with standardized technical standards, refinement of modality specific EPEs, and monitoring of actual EPEs might be needed.

This comparative analysis highlights the importance of ongoing data collection using consistent methods and collaboration between professional organizations and medical physics practitioners. By combining theoretical frameworks with survey data, the medical physics workforce can continuously refine its understanding of workload demands (including time required for EPEs), optimize resource allocation, and improve the quality and safety of patient care across all diagnostic imaging modalities.

There is an important limitation of this analysis: in the 2020 Survey, AAPM Report 301, and the 2004 ACR study, modalities are classified in different subsets which may skew the analysis. For example, AAPM Report 301 has four entries for MQSA mammography surveys, whereas the 2020 Survey has one entry. In these instances, the data were matched as closely as possible (from the 2020 Survey, the MQSA mammography entry is “MQSA‐compliant FFDM with DBT,” which was compared to the entry “MQSA physics survey, DR w/DBT” in AAPM Report 301). These adaptations may lead to inconsistencies in the time comparison, as the complexity of individual EPEs (e.g., a standalone diagnostic CT scanner compared to the attenuation correction CT‐subsystem on a SPECT/CT scanner) may not be adequately captured. This inconsistency was due to the nature of survey question design which must strike a balance between obtaining detailed and meaningful responses and minimizing the time required for participants to complete it, ensuring both the quality of the data and the convenience for respondents. This resulted in the 2020 Survey not reflecting the comprehensive breakdown provided in AAPM Report 301.

### 3.2 Analysis of level 2 services

Level 2 services^3^ encompass a variety of well‐defined responsibilities, tasks, and roles that can be either mandatory or discretionary; some examples of Level 2 services are providing education and training, various radiation, laser, or MRI safety duties, managing policies and procedures, professional service in societies, management of imaging protocols, and patient dosimetry. These services adhere to established methods and standards outlined in national or international guidelines such as AAPM reports, NCRP publications, and NEMA Standards. Medical physicists, owing to their education, training, expertise, and problem‐solving ability, often perform and/or supervise Level 2 services, even though there is no specific requirement or regulation mandating their involvement.

Since Level 2 services are not codified as requirements for medical physicists in regulatory or accreditation programs, it is challenging to determine what fraction of the imaging physics workforce and what percent of full‐time effort (%FTE) of an imaging physicist's workload includes Level 2 services. This difficulty is due to several reasons: some institutions may require a medical physicist to perform Level 2 services whereas others do not; not all institutions will require the same amount of Level 2 services; and depending on local regulations, a Level 2 service in one geographic area (e.g., a state) may be a Level 1 service in another geographic area (e.g., another state). In this report, the LoS model from AAPM Report 301 is used to analyze the roles imaging physicists play in performing Level 2 services.

One key role often occupied by imaging physicists is that of the radiation safety officer (RSO). Table 2 shows 33% of respondents worked as RSO in some capacity. Table 2 further breaks this down into 13%, 27%, and 26% of 2020 Survey respondents serving as RSO as an employee or as a consultant/contractor of the licensee on broad scope RAM licenses, medical RAM licenses, and X‐ray or machine‐source licenses/registrations, respectively. The data do not separate responses and there is overlap for respondents who perform RSO duties in multiple capacities (e.g., one medical physicist may serve in the RSO capacity on a broad scope or medical RAM license as well as an X‐ray machine license). A future survey may need to determine a more precise number of medical physicists serving in RSO roles, particularly as there are projections there will be a requirement for more health physicists in the future.^7^ It is possible that more imaging physicists will be assigned radiation safety responsibilities and roles in the future to address this shortage.

**TABLE 2:** Capacities served as RSO from the 2020 survey. Data from Table 6 of the 2020 survey.

License	Capacity	% of subset of respondents who indicated they serve as RSO	% of all respondents
Broad scope RAM	Employee of licensee	32%	10%
Broad scope RAM license, consultant or contractor	Consultant or contractor	10%	3%
Medical RAM	Employee of licensee	43%	14%
Medical RAM license, consultant or contractor	Consultant or contractor	39%	13%
X‐ray or machine‐source license or registration	Employee of licensee	45%	15%
X‐ray or machine‐source license or registration, consultant or contractor	Consultant or contractor	32%	11%
All license types	Any capacity as RSO	100%	33%

The percentage of imaging physicists who serve as RSO in some capacity differs between AAPM and HPS members as shown in Table 3. A smaller percentage of all AAPM respondents (26%) serve as RSO compared to all HPS respondents (66%) for each RSO capacity, suggesting that HPS members working in the medical field are more likely to work as RSO.

**TABLE 3:** Capacities served as RSO from the 2020 survey for AAPM and HPS members. Data from Table 6a and 6b of the 2020 survey (these tables were generated from the 2020 survey by the AIP and are available upon request).

	AAPM members		HPS members	
Capacity	% of AAPM respondents indicating they serve as RSO	% of all AAPM respondents	% of HPS respondents indicating they serve as RSO	% of all HPS respondents
Broad scope RAM license as employee of licensee	21%	5%	57%	37%
Broad scope RAM license as consultant or contractor	4%	1%	8%	5%
Medical RAM license as employee of licensee	47%	12%	49%	32%
Medical RAM license as consultant or contractor	38%	9%	26%	17%
X‐ray or machine‐source license or registration as employee of licensee	48%	12%	64%	42%
X‐ray or machine‐source license or registration as consultant or contractor	33%	8%	17%	11%
All license types (any capacity as RSO)	100%	26%	100%	66%

*Note*: This table includes data from respondents who reported working full‐time and whose total hours ranged from 30 to 80 h per week.

Tables 2 and 3 demonstrate that an RSO is more likely to be an employee of the facility as opposed to a consultant or contractor; this is especially true for broad scope RAM licenses. Note that the sum of percentages for those indicating they serve as an RSO is greater than 100%; this means that some individuals serve in multiple capacities or on multiple license or registration types as an RSO.

Justification of imaging physicist staffing at an individual facility and estimates of the future demand for imaging physicists must consider the time and effort spent working in RSO capacities. Growth in the demand is reasonably anticipated as more Level 2 radiation safety services are required; this in turn is predicated on the expected growth in overall population,^8^ increase in the patient population for radiological care (estimated from increase in procedures using ionizing radiation^9^), refinements in patient dose calculations from radiological procedures (such as peak skin dose estimates in fluoroscopy^10^), and rapid growth in radiation safety‐intensive services such as theranostics^11^ which can result in increased radiation exposure to the patient, staff, and general public.^12^, ^13^, ^14^ Imaging physicists already contribute substantial effort to radiation safety and can be expected to experience further increases in that share of their time and effort.

The 2020 Survey examined the average number of hours per month respondents who serve as RSOs spend on various radiation safety tasks (Table 4). For an RSO performing all of these tasks, this translates to 13.0 average hours per week and 5.8 median hours per week (0.325 FTE and 0.145 FTE, respectively, for a 40‐h work week) for these regulatory radiation safety requirements. The substantial difference between mean and median reflects the wide variability in RSO workload across respondents, with some individuals dedicating significantly more time to RSO duties. The three most time‐consuming radiation safety tasks identified were calibrations and quality control (including sealed source surveys), audits, and radiation shielding tasks. The three tasks performed by the most respondents were policy or procedure writing, tasks related to the radiation safety committee, and personnel dosimetry program reviews.

**TABLE 4:** Hours per month respondents serving as radiation safety officers (both AAPM and HPS members) spent on various tasks. Data from Table 7 and 7R of the 2020 survey (these tables were generated from the 2020 survey by the AIP and are available upon request).

Activity	Average (hours per month)	Median (hours per month)	Number of respondents
Review of personnel dosimetry or ALARA program	4.9	2.0	247
Auditing of radiation safety program records and documents	8.1	3.0	245
Attending and preparing for Radiation Safety Committee and similar meetings	5.0	2.0	286
Observation and evaluation of clinical personnel radiation safety practices	5.6	2.0	236
Lead apron or person shielding inspection and QC training	3.4	2.0	128
Writing and revising policies, operating procedures, and safety procedures	7.2	4.0	303
Preparation and submission of license or registration applications, amendments, and related regulatory agency correspondence	6.0	2.0	216
Calibration and QC of radiation survey, counting, and measurement instruments including dose calibrators, sealed source inventory or leak testing	8.2	4.0	207
Shielding designs, evaluations, or radiation safety surveys for radiation therapy	7.8	4.0	219

*Note*: This does not include respondents who reported working 0 h in a particular setting. This table includes data from respondents who reported working full‐time and whose total hours ranged from 30 to 80 h per week.

Table 4 shows that the mean and median time spent on well‐defined radiation safety duties for radioactive materials would be about 56 h per month and 25 h per month, respectively, across all respondents for a given respondent who performs all these duties. The comparison in Table 5 shows that HPS member respondents spend considerably more time on these tasks than AAPM members; for all tasks using the average values, HPS respondents spent a median 56 h per month compared to a median 19 h per month for AAPM respondents for a given respondent who performs all these duties. This may indicate that HPS members working in medicine are more likely to work entirely in radiation safety, whereas AAPM members are likely to have more clinical imaging physics duties.

**TABLE 5:** Median hours per month respondents serving as radiation safety officers spent on various tasks. Data from Table 7a and 7b of the 2020 survey (these tables were generated from the 2020 survey by the AIP and are available upon request).

	AAPM		HPS	
Activity	Median hours per month	Number of respondents	Median hours per month	Number of respondents
Review of personnel dosimetry or ALARA program	2.0	184	5.0	63
Auditing of radiation safety program records and documents	2.0	181	10.0	64
Attending and preparing for Radiation Safety Committee and similar meetings	2.0	223	8.0	63
Observation and evaluation of clinical personnel radiation safety practices	2.0	180	5.0	56
Lead apron or person shielding inspection and QC training	1.0	88	2.5	40
Writing and revising policies, operating procedures, and safety procedures	2.0	237	10.0	66
Preparation and submission of license or registration applications, amendments, and related regulatory agency correspondence	2.0	158	5.0	58
Calibration and QC of radiation survey, counting, and measurement instruments including dose calibrators, sealed source inventory or leak testing	3.0	155	5.5	52
Shielding designs, evaluations, or radiation safety surveys for radiation therapy	3.0	168	5.0	51

*Note*: This does not include respondents who reported working 0 h in a particular setting. This table includes data from respondents who reported working full‐time and whose total hours ranged from 30 to 80 h per week.

In the 2020 Survey, respondents were polled on several non‐mandatory Level 2 services (Table 6), which included developing or delivering training, delivering ongoing education, and supervising trainees. Imaging physicists engage in various Level 2 services that encompass extensive training efforts across multiple domains. The nature of these training efforts was not investigated in the 2020 Survey, but we speculate that these efforts likely focus on 1) improving quality control (QC) measures and initiatives and 2) teaching and mentoring future medical physicists and other healthcare professionals (technologists, graduate students, medical students, residents, fellows). These tasks are aimed at enhancing quality improvement within healthcare settings.

**TABLE 6:** Time spent on various tasks by survey respondents. Data from Table 8 of the 2020 survey.

Setting	Measurement span	Average (h)	Number of respondents
Developing or delivering training	Month	4.6	267
Delivering ongoing education	Week	4.8	203
Supervising trainees	Week	5.7	171
Reading and responding to email and similar messaging	Day	2.1	409
Attending meetings and making phone calls	Week	6.0	409
Travelling between work locations (pre‐COVID 19)	Week	4.6	268

*Note*: This does not include respondents who indicated they had not performed the task. This table includes data from respondents who reported working full‐time and whose total hours ranged from 30 to 80 h per week.

There are several activities in Table 6 that cannot be well‐defined as imaging physics specific services but which are still important to consider. The first is that, on average, imaging physicists spend 2.1 h per day reading and responding to email and similar messaging and 1.25 h per day on meetings or phone calls; in total, this represents 42% of an 8‐h working day. This almost certainly includes email, meetings, and phone calls that are part of performing other medical physics services (e.g., a radiation safety committee meeting), but the amount of overlap was not quantified in this survey. This highlights a potential opportunity to improve communication efficiency, as these time demands may contribute to the fact that 92% of respondents reported having chosen to work outside their regular hours, while 82% indicated they were required to do so. The time spent on these non‐mandatory Level 2 services was similar between AAPM and HPS members responding to the survey.

In the 2020 Survey, physicists were asked to select three additional responsibilities that occupy substantial time and effort and to identify how much time was required by those responsibilities monthly. Table 7 provides both the number of respondents and the mean time these respondents spent on these additional responsibilities. The three areas with the most respondents (outliers removed) were clinical quality improvement (127 respondents perform this work, mean 12.2 h per month), support of inspections and surveys by regulatory authorities (101 respondents perform this work, mean 8.2 h per month), and daily or weekly equipment quality control (94 respondents perform this work, mean 11.1 h per month). This suggests that most Level 2 services are still focused on clinical support, and that these occupy a large portion of the imaging physicist's time and effort.

**TABLE 7:** Additional level 2 services provided by imaging physicists with outliers removed. Data from the 2020 survey analysis of respondent selection of three extra duties that take up considerable time and effort.

	Number of respondents performing service	Mean hours per month
Clinical quality improvement	127	12.2
Support of inspections and surveys by regulatory, accreditation, and similar authorities	101	8.2
Daily or weekly equipment quality control	94	11.1
Shielding design, evaluation, surveys	81	11.0
Direct patient care such as support for the therapy or interventional procedures	81	28.1
Professional volunteer service such as leadership roles and committee or task group memberships in local, regional or national organizations	79	16.3
CT protocol review and CT protocol committee	77	7.4
Basic scientific research	77	48.4
Clinical research	72	16.7
Research and development of medical physics tools, techniques, operational improvements	62	30.4
Patient and fetal dose calculations or estimates	54	4.9
Personnel management such as recruiting, hiring, evaluating, development, HR, etc.	47	13.0
MR safety program, MR safety officer and expert duties	44	11.0
Education administration such as program director, program committee member, program committee chair, etc.	36	15.2
Operations management such as client relations, finances, scheduling, etc.	31	18.0
Fluoroscopy, interventional protocol review and fluoroscopy protocol committee	30	13.9
Informatics	22	29.1
Laser safety program, laser safety officer duties^*^	21	10.9
Equipment selection and life cycle management	18	15.3

* In the medical environment, the Medical Laser Safety Officer (MLSO) is the designated authority responsible for the development, implementation, and oversight of the laser safety program. This role is governed by the standards set forth in ANSI Z136.3 Safe Use of Lasers in Health Care. The MLSO's responsibilities include administrative, technical, and operational oversight to ensure the safety of patients, staff, and the environment. Typical LSO duties include maintaining an inventory of Class 3B and Class 4 medical laser systems, ensuring that appropriate safety equipment is procured and used, conducting audits and clinical observations, overseeing laser safety training, developing and implementing laser safety policies and safe operating procedures, and participating in investigations of safety incidents.

Among the selectable options for additional responsibilities, those respondents indicating some form of research as a Level 2 activity were generally associated with higher average time commitments for that activity. This included basic research (77 respondents perform this work, mean 48.4 h per month), research and development of medical physics tools, techniques, and operational improvements (62 respondents perform this work, mean 30.4 h per month), and clinical research (72 respondents perform this work, mean 16.7 h per month). Another Level 2 area that demands substantial effort for the imaging physicists who perform this work is professional volunteer service such as leadership roles and committee memberships in local, regional or national organizations (79 respondents perform this work, mean 16.3 h per month).

The survey results demonstrate that for those imaging physicists who provide Level 2 efforts in direct patient care, including support for therapeutic or interventional procedures, a substantial time effort is provided (81 respondents perform this work, mean 28.1 h per month). Future efforts should evaluate if the number or effort level of imaging physicists providing direct patient care will increase as this may signify a faster‐growing demand for imaging physicists in clinical health care settings. Additionally, as medical technology continues to advance and as the concept of personalized, precision medicine emerges as an expectation, the workload and responsibilities of medical physicists will likely evolve correspondingly. Some examples include the implementation of artificial intelligence (AI) in the clinic, the rapidly expanding field of theranostics, and highly specialized imaging protocols. Medical physicists may not currently be required to work in these areas, but their involvement will be vital both to deliver high quality care and to ensure access for increasing numbers of patients in the future.

Historically, little if any clinical work performed by imaging physicists has been directly reimbursable. There have been recent additions or increased utilization of various current procedural terminology (CPT) codes relevant to imaging physics practice, including CPT Code 76145 (for fluoroscopy peak skin dose calculations),^15^ CPT Codes 76014 and 76015 (MRI safety assessment of foreign bodies or implants),^16^ CPT Code 76017 (for MRI protocol customization by a medical physicist or other MR safety expert to address MR safety concerns),^16^ and CPT codes 77300, 77370, 77261, and 77295 (for various theranostics procedures).^17^ These CPT codes provide a mechanism for improved reporting and reimbursement for Level 2 services that can be provided by imaging physicists. The 2020 Survey did not ask respondents whether the amount of their work, if any, was being reimbursed utilizing these CPT codes or similar mechanisms. However, it is expected that if reimbursement for imaging physics Level 2 services becomes more highly utilized, it will increase the amount of these Level 2 services provided by imaging physicists.

### 3.3 Workforce age and lifestyle factors

Age distribution in the imaging physics field (including AAPM and HPS members) skews heavily toward older professionals. From recent (2025) Labor Force Statistics from the Current Population Survey published by the U.S. Bureau of Labor Statistics (https://www.bls.gov/cps/cpsaat18b.htm), the median age of workers in various healthcare settings ranges from 40 to 47 years with less than 7% of the healthcare workforce 65 years and older. The 2020 Survey showed that the median age in imaging physics was 55 years, with 30% of respondents older than 65 and an equal percentage under 51 years (Figure 1). The distribution shown in Figure 1 raises concerns about a near‐term shortage of mid‐career professionals to assume leadership roles as senior physicists retire.

**FIGURE 1:**
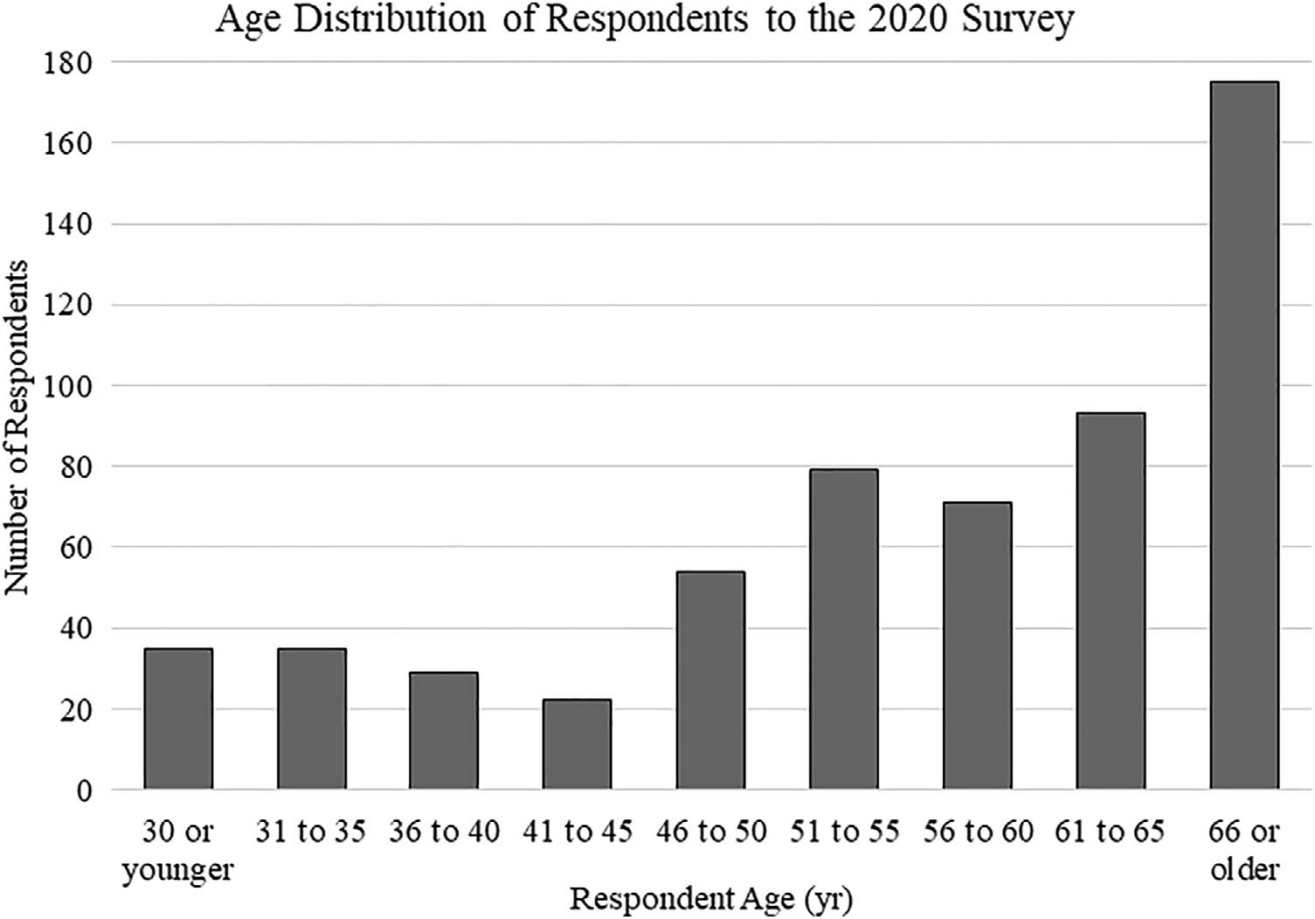
Age distribution of respondents who provided their age in the 2020 survey. Data from Figure 1 of the 2020 survey.

Table 8 provides further insight into these age‐related trends; across all full‐time respondents, the mean age of the imaging physics workforce is 52.5 years, with 75% of the workforce 44 years or older, 50% of the workforce 54 years or older, and 25% of the workforce 62 years or older. These numbers are consistent with 2022 AAPM membership demographics provided by AAPM staff from the AAPM membership database, which show diagnostic imaging physicists have mean age 54.8 years and median age 55 years. For comparison, the average age of therapy physicists was 43.6 years as reported in the 2019 Therapy Workforce Survey (https://www.aapm.org/pubs/surveys.asp). The consistency across these datasets lends credibility to the survey results and reinforces the conclusion that the imaging physics workforce faces a significant challenge as a large proportion of its membership approaches, or has already reached, retirement age. This aging workforce could lead to potential knowledge gaps, skill shortages, and disruptions in operations unless proactive measures are taken to recruit younger individuals to ensure the field continues to thrive.

**TABLE 8:** Age information of 2020 survey respondents working 30 to 80 h per week. There were 383 total respondents and 96 respondents with primary specialty diagnostic radiology to this question.

Number of respondents	Age of all respondents (years)	Age of respondents with primary specialty diagnostic radiology (years)
Minimum (years)	25.0	28.0
Lower quartile (years)	44.0	46.0
Median (years)	54.0	55.5
Upper quartile (years)	62.0	62.0
Maximum (years)	81.0	80.0
Mean (years)	52.5	53.6

*Note*: There is no evidence to suggest a statistically significant difference between the ages reported by respondents whose primary specialty is diagnostic radiology and those with other primary specialties.

Figure 2 and Table 9 detail the career plans of the survey respondents in various age groups. These data show that 35% of the 2020 Survey respondents, including 57% and 78% of the survey respondents age 61–65 years and ≥ 66 years or older, respectively, planned to retire by 2025 or 2026 (this is within 5 years of the 2020 Survey administration). This is concerning as the percentage (35%) of survey respondents planning to retire at that time exceeded the total number of respondents under 50 (30%). These trends align with other observations of rising retirement rates since 2010 as baby boomers age out of the workforce.^18^, ^19^

**FIGURE 2:**
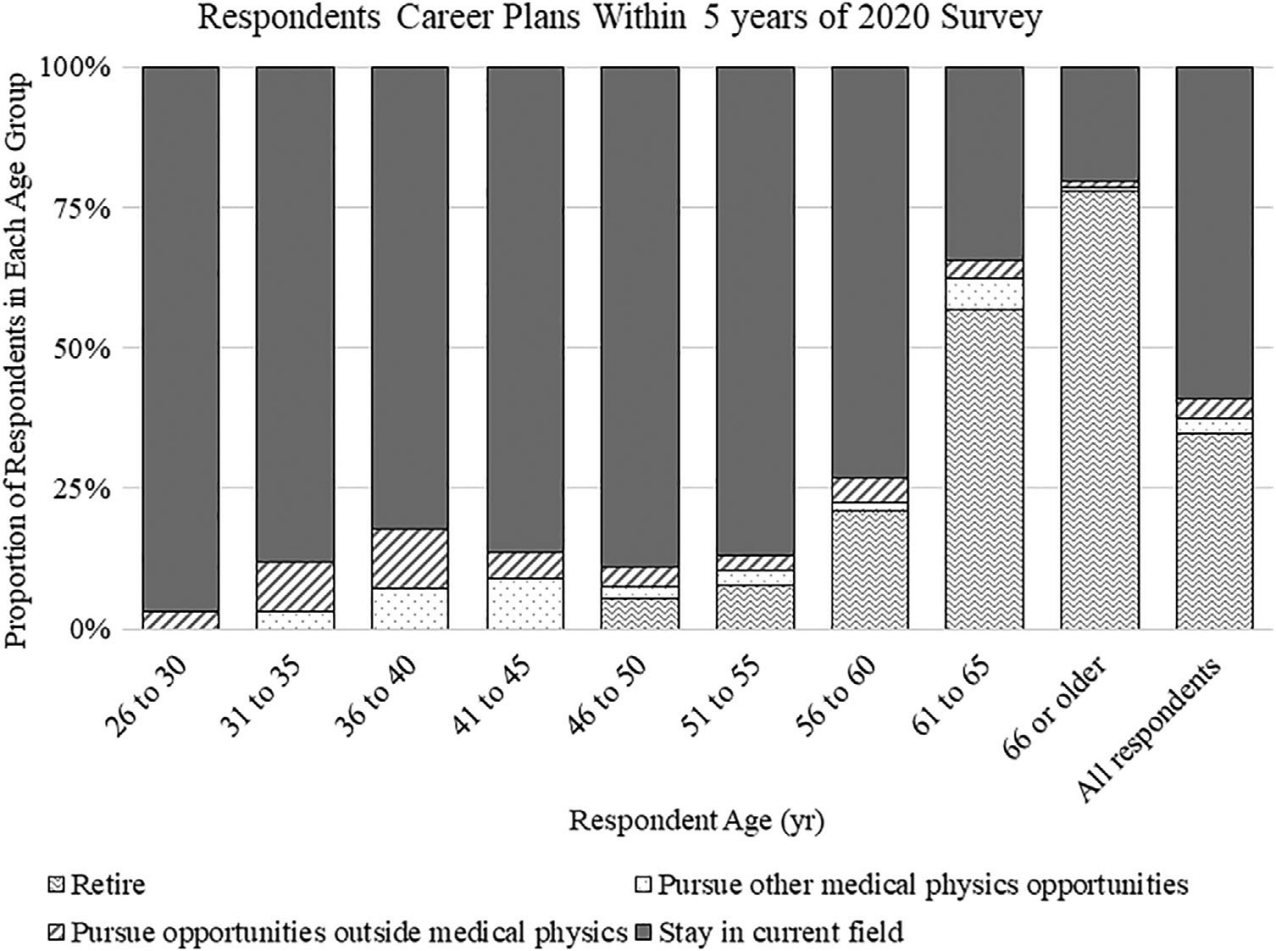
Plans to leave current job by age group from respondents to the 2020 survey. Refer to Table 9 for details on the number of individuals in each age group. Data from Figure 2 of the 2020 survey.

**TABLE 9:** Plans to leave current job by age group from respondents to the 2020 survey who provided this information. Data from Figure 2 of the 2020 survey.

	Number of physicists					
Age group (years)	Retire	Pursue other medical physics opportunities	Pursue opportunities outside medical physics	Stay in current field	Total	% of physicists planning to leave field
30 or younger	0	0	1	31	32	3%
31–35	0	1	3	30	34	12%
36–40	0	2	3	23	28	18%
41–45	0	2	1	19	22	14%
46–50	3	1	2	48	54	11%
51–55	6	2	2	66	76	13%
56–60	14	1	3	49	67	27%
61–65	51	5	3	31	90	66%
66 or older	119	1	2	31	153	80%
All respondents	193	15	20	328	556	41%

In addition to retirement, imaging physicists may also pursue other opportunities within or outside of the medical physics field. Overall, 3% of the 2020 survey respondents (which includes 3% of respondents aged 31 to 35 years, 7% of respondents aged 36 to 40 years, and 9% of respondents aged 41 to 45 years) planned to pursue other opportunities within the medical physics field. The 2020 Survey does not provide a definition of the medical physics field, so it is not clear whether these respondents planned to move to other positions in the imaging physics workforce (e.g., changing institutions) or to leave the imaging physics workforce to work in other areas of medical physics (e.g., radiation oncology, industry, or basic research). 4% of the 2020 Survey respondents (which includes 9% of respondents aged 31 to 35 years, 11% of respondents aged 36 to 40 years, and 5% of respondents aged 41 to 45 years) planned to leave medical physics. This would amount to an annual attrition rate of 1%–2% of the existing imaging workforce in addition to that attributed to retirement.

Those planning to leave their current job may do so for personal and/or professional reasons. The 2020 survey data did not include respondents’ motivations to leave the imaging physics workforce for any of the three alternatives shown in Table 9 (medical physics opportunities outside the imaging physics workforce, opportunities outside of medical physics, or retirement). This information could be queried in a future survey by asking respondents what personal (e.g., travel, long or irregular working hours, moving to a more desirable location, desire to retire, or a desire for change) and professional (e.g., increased compensation and benefits, perceived lack of career opportunities and lower compensation within the workforce, more desirable career opportunities outside the workforce, or burnout from working in healthcare and/or academia) reasons they have for leaving the imaging physics workforce. It may be easier for individuals in imaging physics to change to non‐medical physics careers since their backgrounds vary as shown in Table 10; the highest degrees of only 39% and 16% of the 2020 Survey Respondents to this question were in medical physics or health physics, respectively. These varied academic backgrounds result in unique skill sets that may facilitate career moves into different fields.

**TABLE 10:** Academic discipline of highest degree earned. Data from Table 2 of the 2020 survey.

**Academic discipline**	**Number of respondents**	**Proportion of respondents to this question**
Medical Physics	305	39%
Physics	152	20%
Health Physics	122	16%
Nuclear Engineering	37	5%
Biomedical Engineering	32	4%
Biomedical Physics	24	3%
Electrical Engineering	14	2%
Biomedical Science	8	1%
Some other discipline	82	11%

Table 11 shows the average number of hours worked per week by full‐time respondents to the 2020 Survey in various employment settings. The total time worked by all respondents who reported working full‐time across all settings was 45.3 h per week. All settings in this context represents the average time worked per week per imaging physicist and accounts for imaging physicists working in multiple settings. Imaging physicists worked the fewest hours per week at freestanding clinics, non‐medical colleges and universities, and self‐employed functions. Private or community hospitals, medical physics practices, or consulting groups accounted for the longest average hours worked per week. A key limitation of these data is that only responses indicating total weekly work hours between 30 and 80 h per week were included, thereby excluding some part‐time medical physicists’ responses from these figures. Another limitation is that categories with shorter average work hours are due to individual physicists working in multiple settings, as this survey question allowed individuals to fill in hours for each type of employer (note that all the settings have fewer hours worked per week than the average respondent number of hours worked per week). Thus, Table 11 should not be used to estimate total weekly work time per imaging physicist but may be useful in estimating demand for medical physics services or staffing in various settings. The AAPM also publishes data similar to Table 11 in the members‐only annual AAPM Professional Survey Report (https://www.aapm.org/pubs/surveys.asp). The working hour per week data obtained from the 2020 Survey are either similar to or skew slightly lower than the central tendencies reported in the 2023 AAPM Professional Survey Report. This might be attributed to the age‐skewed distribution of our respondent population, with older individuals phasing‐in retirement by working reduced hours, as well as small sample sizes in some categories. Another confounding difference is that the AAPM Professional Survey Report includes therapy and imaging physicists, whereas the 2020 Survey focused on imaging physicists and those therapy physicists who routinely do imaging work; 77% of the 2023 AAPM Professional Survey Report respondents are therapy physicists, so the AAPM Professional Survey data are skewed towards this specialization and may not accurately reflect the imaging physics workforce. The design of future surveys should consider how to reformulate these questions for more readily interpretable results regarding the imaging physics workforce.

**TABLE 11:** Average number of hours worked per week for each type of employer for those respondents who reported working full‐time. Data from Table 5 of the 2020 survey.

Setting	Average hours per week	Number of respondents
Medical school or university hospital nonclinical duties including teaching and research	37.2	202
Private or community hospital	41.4	154
Government hospital	37.5	19
Government excluding hospitals	36.4	8
Freestanding clinic	28.1	14
Medical practice	38.5	10
Nonmedical college or university	25.4	14
Industrial or commercial firm	39.2	22
Self‐employed in medical physics	22.2	49
Employee or member of medical physics practice or consulting group	41.6	49
Cancer Center	34.9	37
Other	39.3	7
All settings	45.3	476

*Note*: This table includes data from respondents who reported working full‐time and whose total hours ranged from 30 to 80 h per week.

92% and 81% of the 2020 Survey respondents work outside their regularly scheduled hours because they chose to (but were not required to do so) and/or were required to, respectively. Figure 3 shows a relative distribution of the frequency of occurrence and advance notice period reported by a subset of these respondents. Approximately 60% of these respondents were required to work outside their regularly scheduled working hours more than two to three times monthly, and more than half of those who had to work outside their hours more than once per week were notified of the need to do so either the same day or the day before. Most of those who worked outside their regularly scheduled working hours two to three times a month were notified several days to one week in advance. The irregular working hours with short notice may negatively impact work‐life balance and may motivate members of the imaging physics workforce to change jobs or leave the field.

**FIGURE 3:**
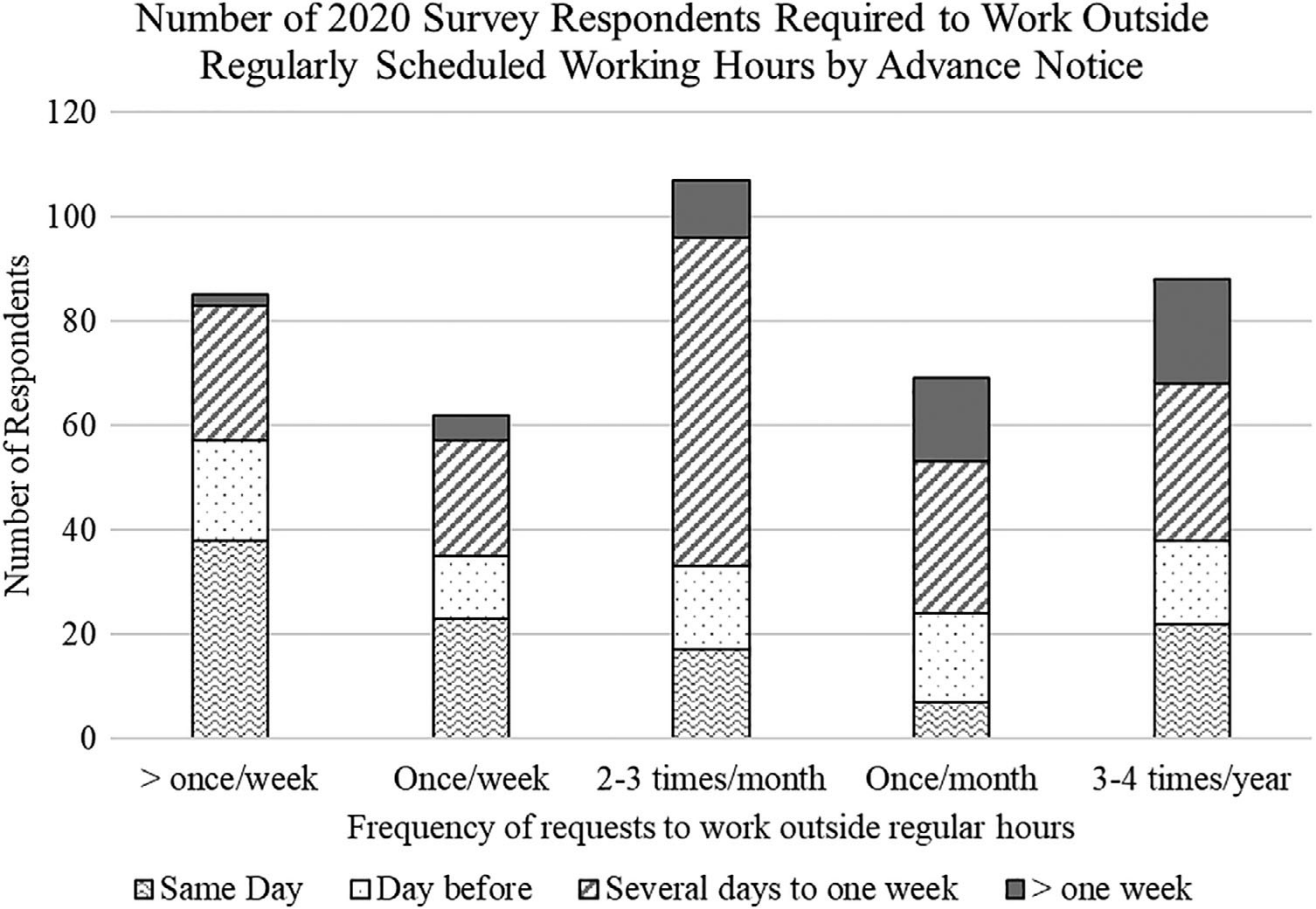
Number of 2020 survey respondents required to work outside regularly scheduled working hours by advance notice. Data from Figure 4 of the 2020 survey.

In summary, up to 3%, 4% and 35% of the survey respondents planned to leave the imaging physics workforce by 2025 or 2026 to pursue other medical physics opportunities, careers outside medical physics, or retire, respectively; the total impact of expected attrition is up to 41% of the 2020 workforce. At the time of this writing (2026), the projected attrition window led by retirement identified in the 2020 Survey has arrived. Currently, comprehensive follow‐up workforce data are not available to validate the projected 42% attrition (35% due to retirement). Characterization of the actual versus predicted attrition remains a critical area for future study and survey follow‐ups.

From other work,^20^ this expected shortage of supply combined with increased future demand is predictive of a persistent workforce shortage. An estimate of the diagnostic imaging physics workforce showed there were approximately 5.5 X‐ray imaging physicists per million U.S. residents in 2019, with approximately 60% of those board‐certified.^20^ This is an increase from an estimated 2.5 imaging physicists per million U.S. residents in 2009,^20^ noting that the limited number of data points may have overstated or inflated this growth rate. This may require more new physicists, who are largely expected to be imaging physics residency graduates, to enter the field to meet demand. Despite being characterized for radiation oncology physics residency programs,^19^, ^21^ an estimate of necessary existing and/or new imaging physics residency positions has not been performed for imaging physics programs.

With this growth in employment need and the expected shortage of incoming physicists and imminent retirements, the gap between supply and demand may widen in the coming years, although it is challenging to identify 1) the total numbers of individuals leaving and entering the imaging physics field and 2) the optimal number of imaging physicists required at any given time in the field. Overcoming both challenges is required to fully characterize the supply versus demand of imaging physicists.

The dynamics of market growth and supply‐demand imbalance highlight the necessity for regular, comprehensive studies to monitor trends in age distribution, retirement plans, and career trajectories. With many imminent retirements, it is important to find ways to pass on knowledge from senior imaging physicists before they retire. This could include mentorship programs, phased retirement options, and additional retention strategies to enhance job satisfaction. It's also worth noting that over a third of imaging physicists in 2019 were not board‐certified^20^; this correlates well with the 2020 Survey where 69% of respondents were board certified in medical or health physics. This may point to potential barriers in alternative pathways during educational programs and certification processes relative to the standard ABR board certification pathway (completing an accredited graduate/certificate program followed by an accredited residency). If board certification becomes more desirable or required by health care institutions or regulations, this could further affect the ability of the imaging physics workforce to keep up with demand. To address the challenges of an aging workforce and potential supply‐demand gaps, the 2020 Survey results indicate the need for leadership development, enhancing alternative pathway diversity, and coming up with new ways to transfer knowledge and keep trained imaging physicists in the field to meet growing healthcare needs.

### 3.4 Skills and knowledge gaps

The results of the 2018 Survey (which asked employer‐related questions to physicists involved in the hiring process) and the 2020 Survey (which only queried demographics and work performed by the imaging physics workforce) demonstrated that perceived and actual skill and knowledge gaps exist, reflected in differences between the data acquired in both surveys. Respondents to the 2018 Survey who played a role in the hiring process were asked to provide the main hiring difficulties they encountered. The four main hiring difficulties identified from this query were: 1) applicants lacked relevant work experience (546 respondents); 2) small numbers of applicants (477 respondents); 3) applicants lacked professional skills, such as organization, professionalism, or interpersonal skills (395 respondents); and 4) applicants lacked technical or occupational skills (383 respondents). Hiring difficulties 1, 3, and 4 are a result of applicants lacking experience and skills; this was the primary hiring difficulty.

Figure 4 shows that among respondents to the 2020 Survey with more than 1 year FTE experience, 21% had ≤5 years, 21% had 6–20 years, and 58% had ≥21 years of total FTE experience. These data demonstrate the workforce is experienced, which contradicts the most common hiring difficulty (workforce lacks experience). Several explanations may account for this disconnect. First, experienced professionals (majority of workforce) are likely established and thus less likely to move into new roles or positions relative to less experienced early‐ to mid‐career individuals, making the applicant pool less experienced relative to the workforce as a whole. Second, there may be a conflation of this hiring difficulty with the second most common hiring difficulty (small number of applicants). If there is a limited pool of applicants, a hiring manager may be forced to consider individuals with lower levels of experience for the role, which may result in the perception that the workforce lacks relevant experience. Finally, given the workforce's overall seniority, any new entrant to the field may appear underqualified due to recency bias.

**FIGURE 4:**
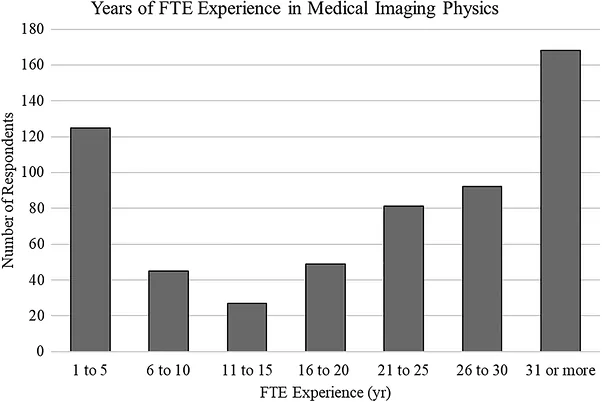
Years of FTE Experience in medical imaging physics. Data from Figure 3 of the 2020 survey.

Table 12 shows 24 areas of skills and knowledge, with the proportion of 2020 Survey respondents who felt well or very well trained for each area identified. All these areas can be relevant during the hiring process depending on the nature and duties of position; depending on the area, the lack of experience and skills in the workforce (applicant pool) is both supported and not supported by the 2020 Survey Data, as discussed below.

**TABLE 12:** Proportion of 2020 survey respondents reporting being trained well or very well in each knowledge/skill. Data from Table 9 of the 2020 survey.

Knowledge/skill	Technical skill (T) or a professional skill (P)	% reporting being trained well or very well
Working on a team	P	95%
Solving technical problems	T	93%
Basic physics principles	T	90%
Performing quality control	T	89%
Variety of equipment or instrumentation	T	84%
Working with customers/clients	P	79%
Managing projects	P	78%
Teaching	T and P	77%
Advanced physics principles	T	76%
Technical writing	T and P	74%
Basic clinical or medical, biology, or radiation biology	T	72%
Public speaking	P	71%
Working with patients	P	69%
Managing people	P	66%
Statistics or advanced math	T	59%
Advanced clinical or medical, biology, or radiation biology	T	54%
Basic research	T	52%
Managing finances or budget	T	50%
Design and development	T	49%
Clinical research	T	46%
Tech support or computer administration	T	40%
Pre‐clinical research	T	37%
Programming or systems software	T	37%
Simulation or modeling	T	33%

Table 12 shows < 50% respondents reporting being well or very well trained in 1) design and development, 2) simulation, modeling, and programming, 3) clinical and pre‐clinical research, and 4) IT support/administration; this supports the 2018 Survey perception that the workforce lacks experience and technical skills. Most of these areas (design, development, simulation, modeling, programming, and IT) are technical and in general keep up with recent advances in computational hardware, software, and theory. Open positions requiring experience in these technical and current areas are likely suffering from a lack of qualified applicants. The other area demonstrating a lack of experience is clinical and pre‐clinical research. This is not surprising; while medical physicists entering the field are currently required to have graduate training prior to residency, there is no requirement for clinical or pre‐clinical research experience (or even research related to medical physics, in the case of the CAMPEP certificate pathway). Additionally, as most residency programs are two‐year long clinical programs (as of 2023), there may be little, if any, time for residents to gain further experience in these research areas. If employers feel physicists who have recently completed their training are not sufficiently trained in clinical and pre‐clinical research, this would represent a disconnect between the preparation provided by graduate and residency programs and the expectations that employers have of new hires.

However, Table 12 also shows a ≥ 50% of 2020 Survey Respondents felt well or very well trained in 18 of the 24 knowledge and skills areas, including physics, math, medical, and biological knowledge, problem solving, performing equipment QC, working with a variety of equipment, technical writing, and basic research. These are aligned with typical imaging physicist duties, but are not aligned with the hiring manager perception of an untrained workforce, which is a disconnect from the 2018 Survey. This discrepancy might be due to self‐bias among 2020 Survey respondents, who may perceive themselves as well trained, while hiring managers from the 2018 Survey might not share this view. The 2018 Survey's open‐ended format also left it unclear which specific skills were perceived as deficient. Additionally, differences in respondent degree types between surveys may contribute to the disconnect, though degree‐level analysis was not feasible given small subgroup sizes (e.g., 15 DMP respondents). A future survey could be designed to directly address these gaps.

One additional item of note regarding relevant experience is that prior to 2014, the ABR did not require completion of an accredited residency program to obtain board certification; indeed, due to age and experience distribution of the field, this resulted in only 7% of 2020 Survey respondents having completed an imaging physics residency (with an additional 1% currently in an imaging physics residency program). During the period immediately preceding the 2018 survey, there may have been early‐career imaging physicists entering the workforce directly from graduate programs without residency training. If the graduate program did not include a strong clinical imaging component, this would result in hiring managers identifying a lack of relevant work experience. It may be useful to re‐survey hiring managers to identify whether early career applicants now have more relevant experience than applicants prior to 2018; if the majority of early career applicants are residency graduates, who should be well‐trained in clinical imaging physics skills,^6^ it is possible that the disconnect regarding experience between the hiring managers and the workforce has been reduced. It may also be useful to survey hiring managers on the demographics of their typical applicant pool. This would test the hypothesis that imaging physics residency graduates possess sufficient relevant work experience and could also identify how often applicants with experience other than imaging physics are applying for these positions. It is possible that the current imaging physics training paradigm produces sufficiently experienced physicists to join the workforce but that it is simply not producing enough of them, which is why other less qualified applicants are considered and hired. This would substantially change the focus to inadequate supply (quantity) rather than inadequate training (quality).

Table 12 shows ≥ 66% of 2020 Survey respondents felt well trained or very well trained in all professional skills identified. This contrasts with the 2018 Survey perception that the workforce lacks professional skills, and may again be due to workforce self‐bias. A follow‐up survey may be useful to identify specific professional skills hiring managers believe applicants lack. Similar to our previous speculation on the recent impact of residency requirements, CAMPEP has recently included some professional skills (e.g., professionalism, leadership, ethics) in the residency curriculum standards; individuals who completed programs before these were required could contribute to the perceived lack of professional skill training. Further training in such professional skills could be implemented through changes to the recommended graduate or residency program curricula or through offering programs through the AAPM (such as Medical Physics Leadership Academy) or other organizations.

It is possible that the experience and skills indicated as lacking in Table 12 could be provided by graduate and residency programs. For example, a weakness self‐identified by the 2020 Survey respondents was lack of advanced computational training (e.g., simulation, modeling, programming); these could be addressed in programs with dedicated training requirements (graduate or residency) but would be much more difficult to acquire while working full‐time as a clinician.

Indeed, there may be many opportunities to improve the training of future imaging physicists. Table 6 indicates that 267 respondents developed or delivered training, spending an average of 4.6 h per month on this task. Additionally, 171 of the respondents supervise trainees, and the average time spent on this supervision is 5.7 h per week. Noting that not all the training materials or time spent supervising went towards medical physics students and medical physics residents, this may further support the apparent lack of experience and skills by the workforce; there were simply not enough trainers and/or hours dedicated to conducting training.

The other primary hiring difficulty identified in the 2018 survey was a small number of applicants, which can impact the perception of applicants lacking skills. If there were few applicants relative to open positions, applicants may be less inclined to improve their technical and professional skills as they would not be competing for employment, and employers may be more inclined to consider applicants with substantial skill deficiencies. Based on the 2020 Survey results, this hiring difficulty may be challenging to overcome.

In Figure 4, the overall trend is a large proportion of physicists with both relatively low levels of experience (1 year < FTE experience ≤ 5 years) and high levels of experience (≥ 21 years FTE experience). Only 16% of respondents to this question had between 5 and 21 years FTE. With the potential attrition rates discussed above, this suggests that the number of new physicists entering the field is increasing, although it is not clear why the proportion of physicists with mid‐career levels of experience (> 5 years FTE and < 21 years FTE) is so low and with high levels of experience (≥ 21 years FTE) is so high. One possible explanation for this may arise from the data in Figure 1 which shows the age distribution of the survey respondents. Figure 1 shows fewer survey respondents were aged 36 to 40 years and 41 to 45 years compared to other age brackets; individuals of these ages would be likely to have mid‐career levels of experience (> 5 years FTE and < 21 years FTE). Table 13 shows this trend of fewer survey respondents in the 36 to 45 years does not match the results for the 2019 Therapy Workforce Survey (https://www.aapm.org/pubs/surveys.asp), which randomly sampled the AAPM membership at the same time. This discrepancy lends credence to the apparent lack of mid‐career imaging physicists. However, a limitation of this work is that the age distribution of AAPM and HPS membership was not collected at the time of the survey, and so the possibility exists this age demographic under‐responded which accounted for the apparent lack of mid‐career imaging physicists.

**TABLE 13:** Relative age distribution (% of total respondents) of 2020 survey respondents relative to the 2019 therapy workforce survey (https://www.aapm.org/pubs/surveys.asp). The AIP divided the AAPM membership randomly into two equal survey lists, with one list used for the 2020 survey and the other list used for the 2019 therapy workforce survey.

Age group (years)	DWS 2020 survey respondents	Age group (years)	TWS 2019 survey respondents
≤35	11.8%	< 35	14.0%
36–45	8.6%	35–44	47.0%
46–55	22.4%	45–54	25.0%
56–65	27.7%	55–64	10.0%
≥ 66	29.5%	≥65	4.0%

The 2018 and 2020 Surveys do not provide data to compare the rate of imaging physicists entering the field with the expected large number of upcoming retirements. More data are required to determine how many qualified applicants are graduating from accredited residency programs to determine if the anticipated attrition rate will be matched by physicists entering the field from traditional means. There is an apparent residency bottleneck; residency training is currently required for ABR certification, but each year only 60%–70% of applicants successfully secure residency program training positions through the MedPhys Match offered through the National Matching Services Inc (https://natmatch.com/medphys/statistics.html). It is important to note that these data are aggregated across all types of residency programs (therapy, diagnostic, and nuclear medicine) and are incomplete. Not all residency programs participate in the MedPhys Match; some applicants registered in the Match may accept positions in non‐Match programs which inflates the number of apparentlyunmatched residency Match applicants. Additionally, not all prospective residents utilize the MedPhys Match and may only apply for and accept residency positions outside the MedPhys Match. More complete data on imaging physicists entering the field may be obtained from CAMPEP (https://campep.org/campeplstres.asp), which compiles program statistics for all accredited residency programs which serve as one of the major pipelines of new imaging physicists into the workforce. In future models and analysis of individuals entering the imaging physics workforce, these statistics will be requested from CAMPEP. A limitation of this current work is that these CAMPEP data was not obtained and used.

When evaluating skills and knowledge gaps, it is important to identify any skills that were missing or under‐represented in the reported 2020 Survey when cross analyzed with the 2018 Survey. Table 12 (2020 Survey) appears comprehensive and there are no obvious omissions when compared to the less granular 2018 survey data. A follow‐up survey would be required to identify whether the imaging physics workforce is lacking any critical skills not identified in the current surveys, perhaps by providing a free‐form field in addition to listed skills.

It is difficult to predict how some of the reported skills and knowledge in the 2020 Survey will align with future requirements for medical physicists as this was not queried with the recent survey data. More data could be collected to evaluate the preparation of the imaging physics workforce for emerging practices that may become standard in the near future, such as AI, theranostics, imaging technology advances (e.g., photon counting CT), changes in regulatory and accreditation requirements, and expanded use of imaging in surgery, therapeutics, and other areas of medicine.

The findings regarding skills and knowledge gaps may suggest shifts in medical physics education. For example, this information may be useful in evaluating graduate program curricula and degree paths, such as whether fewer or more MS, PhD, certificate, and DMP programs are needed. It may also inform undergraduate students and others interested in medical physics with accurate expectations of the education required to be competitive in the field for residency and career positions. The knowledge and skills possessed by the current workforce could be used as a basis for building the future of the field.

## 4 CONCLUSIONS

The 2020 Survey provides updated estimates for EPE time estimates and validates those detailed in AAPM Report 301, accounting for methodological differences concerning task scope and modality classifications. Advanced imaging modalities require more time due to their complexity and stringent compliance needs. These time requirements were relatively stable for over 15 years. Variations in modality classifications and survey design introduce potential limitations to comparative analysis and should be addressed to ensure consistency in data and interpretation in future studies.

The 2020 Survey data indicate substantial imaging physicist involvement in RSO duties across various license types. Imaging physicists also dedicate substantial time to clinical training and quality improvement, which enhance healthcare delivery and patient safety. Emerging trends imply evolving responsibilities for imaging physicists. These trends and the potentially growing demand for patient safety and radiation protection suggest a need for continued workforce assessment and adaptation to ensure that the field continues adapting in order to remain integral to advancing precision medicine. Follow‐up surveys could provide deeper insights into the frequency and scope of these services, informing future workforce planning and resource allocation.

The medical imaging physics workforce is aging, with 30% of professionals over 65 years and a mean age of 52.5 years, raising concerns about a shortage of mid‐career professionals to assume leadership roles. The 2020 Survey suggests that 35% of the workforce planned to retire by 2025 or 2026, outpacing new hires and exacerbating potential knowledge gaps and skill shortages. Despite modest growth in residency and graduate programs, it is not clear whether the supply of new imaging physicists will be sufficient to meet demand. This underscores the need for mentorship programs, leadership development, and strategies to retain talent, ensuring continuity in meeting healthcare demands.

The imaging physics workforce faces significant lifestyle challenges and changes, with retirement being a primary consideration due to its older demographic; more than 78% of respondents aged 66 or older in the 2020 Survey planned to retire soon. Many imaging physicists contemplated leaving the field altogether. Working conditions including irregular hours, short‐notice responsibilities, and extensive travel impacting work‐life balance raise concerns about the ability to retain current imaging physicists and attract new ones to the specialty. Although average weekly work hours vary across employment settings, with some offering lighter workloads, the strain of travel and irregular hours poses challenges.

The 2018 and 2020 surveys highlight skill and knowledge gaps within the imaging physics workforce, evidenced by disconnects between hiring manager expectations and workforce self‐assessments. The 2018 employer survey identified major hiring difficulties, including a lack of relevant work experience, professional skills, and technical skills among applicants. These concerns were echoed in the 2020 workforce survey, where 21% of respondents with > 1 year FTE experience had ≤ 5 years FTE experience, contributing to the perception of inadequate experience. Additionally, as the workforce is aging, there is a limited mid‐career pipeline to fill future leadership roles. The 2020 Survey revealed areas where respondents felt undertrained, such as design and development, simulation, programming, and clinical research. Despite most respondents feeling well‐trained in foundational and clinical skills, hiring managers continue to report gaps, potentially due to misaligned expectations, insufficient graduate and residency training in advanced technical skills and professional skills, or a lack of qualified applicants due to supply shortages of trained imaging physicists. Addressing these gaps may require adjustments to educational curricula, expanded residency programs, and targeted workshops. Future surveys could clarify specific employer needs, assess readiness for emerging technologies, and help guide educational strategies to ensure alignment with evolving field requirements.

Finally, a potential limitation of the work is that the survey was administered during the fall of 2020, which was the approximate mid‐point of the COVID‐19 pandemic disruptions to routines for US residents. There are no published studies on how the COVID‐19 pandemic affected imaging physics workforce in the United States (the 2020 survey only asked respondents about their travel time prior to the pandemic as reported in Table 6). A discussion on how the results of the 2018 and 2020 surveys changed as a function of the pandemic is therefore not included in this manuscript as such a discussion would be speculative and not supported by the data from either survey. The results of the 2018 and 2020 surveys provide a much more recent snapshot of the imaging physics workforce than any previously published work^1^, ^2^ and may help guide future work in evaluation of the imaging physics workforce in the post‐pandemic age. More work is needed to identify the extent of the effect of COVID‐19 (and other socioeconomic changes) on the current imaging physics workforce.

## AUTHOR CONTRIBUTIONS

David Jordan, Dustin Gress, Joshua Wilson, Lei Qin, and Ching‐Mei Feng conceived and administered the 2020 Survey. All authors contributed to formal analysis and discussion of the 2020 Survey results, which was compiled into the original manuscript by Joseph R. Steiner. All authors reviewed and edited the manuscript at all stages in the drafting and review process. Figures and tables were produced by Joseph R. Steiner, Shengwen Deng, Megan E. Lipford, Joshua M. Wilson, Lei Qin, and Ching‐Mei Feng, and were subsequently reviewed by all authors. All authors approved the final version of the manuscript.

## CONFLICT OF INTEREST STATEMENT

The Chair of the Diagnostic Workforce Subcommittee (DWWSS) has reviewed the required Conflict of Interest statement on file for each member of DWWSS and determined that disclosure of potential Conflicts of Interest is an adequate management plan. The members of the DWWSS listed below attest that they have no potential Conflicts of Interest related to the subject matter or materials presented in this document: Joseph R. Steiner, Shengwen Deng, Ching‐Mei Feng, Megan E. Lipford. The members of the DWWSS listed below disclose the following potential Conflict(s) of Interest related to subject matter or materials presented in this document: Joshua M. Wilson (license agreement, Gammex), Mary Ellen Jafari (Member of Board of Directors, ABMRS), Lei Qin (Subcommittee Chair, ISMRM), Dustin Gress (employed by ACR), David Jordan (Board Member and Chair, ABMRS; Committee Chair, ACR; Vice President, Brain Health Alliance).

## Data Availability

The AIP summary of the 2020 Survey results and the survey questionnaire are archived on the AAPM members‐only website (https://www.aapm.org/pubs/surveys.asp). Additional data utilized in this report such as the 2018 Survey data are available from the corresponding authors upon reasonable request.
